# Brief Report: Evaluating the Diagnostic Yield of Commercial Gene Panels in Autism

**DOI:** 10.1007/s10803-021-05417-7

**Published:** 2022-01-07

**Authors:** Fiana Ní Ghrálaigh, Ellen McCarthy, Daniel N. Murphy, Louise Gallagher, Lorna M. Lopez

**Affiliations:** 1 Department of Biology, Maynooth University, Maynooth, Co Kildare, Ireland; 2 Department of Psychiatry, Trinity College Dublin, Dublin, Ireland

**Keywords:** Autism, Panel, Sequencing, Genomics, Utility

## Abstract

Autism is a prevalent neurodevelopmental condition, highly heterogenous in both genotype and phenotype. This communication adds to existing discussion of the heterogeneity of clinical sequencing tests, “gene panels”, marketed for application in autism. We evaluate the clinical utility of available gene panels based on existing genetic evidence. We determine that diagnostic yields of these gene panels range from 0.22% to 10.02% and gene selection for the panels is variable in relevance, here measured as percentage overlap with SFARI Gene and ranging from 15.15% to 100%. We conclude that gene panels marketed for use in autism are currently of limited clinical utility, and that sequencing with greater coverage may be more appropriate.

**Supplementary Information:**

The online version contains supplementary material available at 10.1007/s10803-021-05417-7.

## Introduction

The benefits of a genetic diagnosis of autism are extensive (“Genetic Testing Statement | ISPG—International Society of Psychiatric Genetics”). The International Society of Psychiatric Genetics propose in their consensus statement on genetic testing that the “*identification of known pathogenic variants may help diagnose rare conditions that have important medical and psychiatric implications for individual patients and may inform family counselling*”. A genetic diagnosis of autism may allow for the prospect of genetic counselling for affected individuals and their families; it may also provide an affected individual with opportunity to take part in targeted research or receive anticipatory medical advice.

Genetic diagnosis in autism is limited by the ability to robustly determine the relevance of putatively pathogenic genetic variation. Genomic research in autism is progressing quickly, enabled by advancements in next-generation sequencing technologies and the subsequent establishment of large-scale sequencing cohorts and pedigree-based sequencing cohorts (Glahn et al., 2019; Ní Ghrálaigh et al., 2020). To date, many genes have been identified as having some link to autism (Satterstrom et al., 2020). The Simons Foundation Autism Research Initiative (SFARI) Gene database (Abrahams et al., 2013), collates more than 990 genes for which there is evidence of association with autism, however the clinical utility of this database is limited by the absence of systematic curation of gene-condition relationships (Schaaf et al., 2020). Despite this progress in autism genomic research, major challenges remain in the development of targeted gene panels with substantial clinical utility in autism.

At case-level, gene discovery is complicated by the nature of autism as a complex condition with a large degree of phenotypic heterogeneity. A candidate pathogenic variant may be evaluated, in the majority of autism cases, as being contributory to the genetic risk rather than being wholly causative of an individual’s condition. At cohort-level, studies discovering “autism genes” are compounded by an apparent lack of specificity to autism. For example, in individuals affected by both autism and intellectual disability, genes identified show relevance to both autism and other neurodevelopmental disorders (Myers, Challman, Bernier, et al., 2020). For these reasons, the development of effective gene panels to aid autism diagnosis is extremely complicated.

Despite these limitations, commercial gene panels are available and marketed for use in autism diagnosis. Hoang et al. (2018) evaluated many of these gene panels, clearly demonstrating their heterogeneity (Hoang et al., 2018). Their survey shows large variability in the number of genes being tested by panels, lack of consensus in the genes selected for inclusion, as well as variability in the reporting of laboratory qualification and reporting protocols.

## Methods

### Identifying Autism Gene Panels

Gene panels marketed for use in autism were identified and collated through the following approaches: web browser search (search terms *“autism gene panel”*, *“ASD gene panel”*, *“sequencing tests for autism spectrum disorder”*, *“gene panels for autism testing”* and *“autism genetic testing”*), gene panels analysed by Hoang et al. (2018) (Hoang et al., 2018) and Genomics England PanelApp (search terms *“Autism”, “ASD”*) (Martin et al., 2019). Panels identified for which gene lists were not provided were excluded from analyses (CGC genetics “Autism” panel & Michigan Medicine “Autism/ Intellectual Disability Panels”). Gene list sources are outlined in Supplemental Table 1 (collated October 2020-January 2021).

### Refining Gene Lists

Each gene panel identified provides a list of genes targeted by the probes. By nature, these gene lists arise from a variety of sources and were compiled at varying times. For this reason, gene lists were run through HUGO Gene Nomenclature Committee (HGNC) Multi-Symbol Tool (Version: 2021–01-06 update). Where the gene symbol reported by the provider is an approved gene symbol in HGNC, it is used in analyses. Where the gene symbol is no longer approved by HGNC, it was updated to the approved gene symbol given by HGNC. A small number of deviations occurred that could not be resolved, which resulted in the removal of genes from the analyses. The resulting refined gene lists are provided in Supplemental Table 2. Gene counts reported in Table 1 also reflect these updates.

### Estimating Percentage Overlap with SFARI Gene

To determine the relevance of genes targeted in autism, each panel was assessed for the proportion of genes covered that are included in the SFARI Gene database (all gene scores and genetic categories) of genes implicated in autism susceptibility (Version: 2021–01-13 release). Where necessary, the SFARI gene list (n = 1,003) was updated to HGNC approved gene symbols (n = 5) and genes with symbol mismatch (n = 3) were removed. The number of genes targeted by each panel that are included in SFARI gene are presented in Table 1 as a percentage of the total genes in the panel. SFARI Gene was subset to high-confidence autism associated genes, assigned as such based on SFARI gene scoring of 1 or 2. Percentage overlap was again calculated on this subset and presented in Table 1.

### Selection of Clinically Relevant Variants

Clinically relevant variants, as identified and characterised by whole exome sequencing in the Simon’s Powering Autism Research Knowledge cohort, were used to determine the clinical utility of each panel. Variants included in our analyses are those reported in Feliciano et al. (2019) data set 10 (Feliciano et al., 2019), comprising inherited and de novo single nucleotide variants (SNVs), insertion deletion variants (indels) and copy number variants (CNVs). Reported chromosomal abnormalities were not included. Gene lists were assembled to include those for which clinically relevant SNVs and indels could be defined and those that fall within the boundaries of clinically relevant CNVs. While targeted gene panels lack the ability to define copy number variant boundaries, genes within these variants will appear as deleted or duplicated, thus variation will be detected.

### Determining and Reporting Diagnostic Yield

Diagnostic yield was determined by cross-referencing the gene list of each gene panel with the lists of implicated genes in the SPARK cohort. Diagnostic yield was calculated as the proportion of individuals sequenced, for which a relevant genetic variant was identified, corresponding with the genes contained on each gene panel. The number of individuals in the cohort was taken as 472 affected individuals (465 offspring and 7 parents) as detailed by Feliciano et al. (2019). In keeping with this study*,* 13 individuals, those in families self-reporting a genetic diagnosis were not included in the estimates of diagnostic yield. With this justification, diagnostic yield was calculated as the number of individuals with a relevant variant, as a percentage of the total cohort of 459 affected individuals without a genetic diagnosis.

The number of individuals for which a clinically relevant finding would have been identified by using each targeted gene panel is reported for both pathogenic and probable pathogenic variants, as assigned by Feliciano et al. (2019) (Table 1).

### Determining and Reporting Correlation

Pearson’s product-moment correlation was computed with n = 16 degrees freedom for diagnostic yield and number of genes targeted and for diagnostic yield against percentage overlap with SFARI gene (all genes).

## Results

Here we estimate the clinical utility of commercial gene panels marketed for use in autism. Diagnostic yield, which is the proportion of cases interrogated for which a genetic cause can be determined, is a strong measure of the clinical utility of a sequencing technology. Feliciano et al. (2019) estimate the diagnostic yield of whole exome sequencing to be 10.4% in the initial 457 families enrolled in the Simons Powering Autism Research (SPARK) cohort (Feliciano et al., 2019). A ‘likely pathogenic’ variant was identified in a further 3.4% of families studied. This estimate comes from the identification of a variant that fulfils either the ‘likely pathogenic’ or ‘pathogenic’ criteria, according to American College of Medical Genetics and Genomics (ACMG) standards (Richards et al., 2015).

Gene panels relevant to autism are presented in Table 1. To determine the clinical utility of each autism gene panel, variants meeting ‘likely pathogenic’ or ‘pathogenic’ criteria in the SPARK cohort can be limited to those within the gene set of each panel, respectively. In doing so, we ask how many of the pathogenic variants identified by Feliciano et al*.* would have been identified in the SPARK cohort with application of an autism gene panel, instead of application of whole exome sequencing. The diagnostic yield of each gene panel, estimated with respect to Feliciano et al*.* analyses, is presented in Table 1. The diagnostic yields range from 0.22% to 10.02%, with most gene panels achieving a diagnostic yield below 3%.

Gene discovery in autism is ongoing. Most genes included in the commercial gene panels are autism relevant. This is illustrated by the inclusion of a large proportion of the panel-specific genes in the SFARI Gene database (Abrahams et al., 2013)(Table 1). Gene selection for inclusion in autism panels is variable in relevance, ranging in overlap with SFARI Gene from 15.15% to 100%. Diagnostic yield of the gene panels and size of the panel were found to be positively correlated, (r = 0.82, p = 3.033e-05), indicating an increased number of genes per gene panel enables detection of a clinically relevant variant in a greater number of individuals. No significant correlation between percentage overlap with SFARI Gene and diagnostic yield was detected.

**Table 1:** Diagnostic yield of gene panels marketed for use in autism

Service provider	Panel name	Number of genes targeted	Percentage overlap with SFARI gene		Diagnostic yield in SPARK
			SFARI gene All genes	SFARI high confidence Genes (Scores 1 and 2)	
Ambry Genetics	AutismNext Panel	72	87.5%	76.39%	2.61%
Asper Neurogenetics	Autism Spectrum Disorders NGS Panel	76	88.16%	71.05%	2.83% (0.22%)
Blueprint Genetics	Autism Spectrum Disorders Panel	75	45.33%	36%	1.53% (0.44%)
Center for Human Genetics	Autism Spectrum Disorder 53-Gene Panel	53	84.91%	45.28%	1.96% (0.22%)
Centogene	Syndromic Autism Gene Panel	50	88%	76%	2.4% (0.22%)
Centogene	Intellectual Disability Panel	599	43.41%	24.54%	5.23% (1.31%)
EGL Genetics	Autism Spectrum Disorders Tier 2 Panel	62	74.19%	66.13%	2.18%
Fulgent Genetics	Autism NGS Panel	121	76.86%	55.37%	4.36% (0.44%)
GeneDx	Autism/ID Xpanded Panel	2641	20.64%	10.98%	10.02% (3.49%)
GENETAQ	Autism	27	92.59%	66.67%	1.53%
Genomics England PanelApp	Autism (Version 0.20)	733	100%	42.7%	7.63% (1.96%)
Greenwood Genetic Centre	Syndromic Autism Sequencing Panel	83	80.72%	69.88%	3.05%
GX Sciences	Developmental Nutrigenomic Panel	33	15.15%	0%	0.22%
MNG Laboratories	Comprehensive Disability/Autism Panel	1345	19.85%	12.04%	6.1% (1.3%)
Munroe-Meyer Institute	Autism/Intellectual Disability/Multiple Anomalies Panel	117	55.56%	41.88%	2.4% (0.22%)
Prevention Genetics	Autism Spectrum Disorders Panel	170	95.29%	90.59%	6.32% (0.44%)
Reference Laboratory Genomics	Autism Spectrum Disorders (Expanded Panel)	77	77.92%	64.94%	3.05% (0.44%)
Sema4	Comprehensive Autism Spectrum Disorders Panel (228)	228	57.46%	43.42%	4.79% (0.87%)

Presented are gene panels relevant to autism. The number of genes present in each gene panel are correct as of January 2021. Gene lists provided at the sources listed in Supplemental Table 1 were updated to HGNC approved gene symbols where necessary. Percentage overlap with SFARI is estimated as the proportion of genes within each respective gene list appearing in SFARI Gene (01–13-2021 release). This overlap is presented for both the complete SFARI Gene gene lists and the High Confidence SFARI genes only (Scores 1 and 2). Diagnostic yield is estimated as the number of individuals for which a genetic cause of autism was identified as a proportion of those investigated (459 affected individuals for which no genetic diagnosis was previously reported). Pathogenic variation is considered as variants listed in Feliciano et al. (2019). Variants considered are de novo and inherited single nucleotide variants, insertion-deletion variants and copy number variants. Diagnostic yield of pathogenic variation is listed, with the additional diagnostic yield achieved by inclusion of probable pathogenic variants listed in brackets alongside

## Discussion

Considering the low diagnostic yield of the gene panels that were investigated, we can infer that, while the gene selection for inclusion in autism gene panels is evidence-based, these gene lists are not extensive enough to justify use in autism diagnosis, a complex trait for which hundreds of genes have been associated. Critically, if the application of a targeted gene panel to an affected individual’s genome returns negative for pathogenic variation, one cannot conclude that a causative variant is not present. Rather, it is more likely that genetic causes have been missed due to the limited application of the gene panel.

The GeneDx “Autism/ID Xpanded Panel” represents the autism gene panel with the highest number of individuals for which a genetic diagnosis would have been obtained with its application (10.02%). This diagnostic yield is comparable to that of whole exome sequencing, 10.4% (Feliciano et al., 2019) and that of chromosomal microarray sequencing with a median diagnostic yield of 8.1% (Savatt & Myers, 2021). However, important to note is that this gene panel targets many more genes (n = 2,641) than some of the smaller gene panels, for example GENETAQ “Autism” panel (n = 27), with a diagnostic yield of just 1.53%. The positive correlation of diagnostic yield associated with inclusion of a larger number of genes, reflects well the complex genetic architecture of autism and the number of loci expected to be associated. This raises the question whether autism is an appropriate candidate for the development of commercial gene panels, that are reliant and limited due to the size of the gene panel, the cost and current knowledge of the genetic basis of autism, and questions whether developments should focus on application of sequencing technologies with a broader coverage, such as whole genome sequencing. Expanding beyond targeted autism genes, whole genome sequencing presents the opportunity to explore more of the human genome and, ultimately, to further increase the diagnostic yield in autism (Yuen et al., 2015). Progress in non-coding variant annotation and interpretation, accompanied by a decrease in sequencing costs, may further popularize the clinical use of whole genome sequencing. Currently, whole exome sequencing is proposed as the first-tier diagnostic test for neurodevelopmental disorders (Srivastava et al., 2019). The diagnostic yield in autism using clinical exome sequencing has been estimated at 6.1% in autism (20% overall yield in neurodevelopmental disorders) (Martinez-Granero et al., 2021). Genotyping chips have limited clinical utility for rare genetic variation of SNVs and should not be used to guide health decisions without validation (Mn et al., 2021). Autism genetic testing as minimal as cytogenetic microarray and Fragile X testing alone may be all that is feasible in a clinical setting, which is currently the situation in Ireland.

Provided the relevant expertise and infrastructure for variant interpretation are available and cost effective, gene panels have potential for clinical utility. However, current evidence does not support their applicability in autism (Buxbaum et al., 2020; Myers, Challman, Martin, et al. 2020). Achieving the ultimate goal of a comprehensive autism gene panel will require uniform robust phenotyping to account for the heterogeneity in autism presentation. Application of a formal evidence-based gene curation framework, such as that proposed by Schaaf et al*.* (Schaaf et al., 2020), would account for the degree of certainty in autism diagnoses in studies reporting association and account for co-morbid diagnoses, providing consistency throughout gene discovery. To conclude, evaluation of the diagnostic yield of commercial gene panels marketed for autism determines that they are currently of very limited clinical utility.

## Supplementary Information

Below is the link to the electronic supplementary material.

Supplementary file1 (CSV 2 KB)

Supplementary file2 (CSV 82 KB)

## Data Availability

All data generated or analysed during this study are included or referenced in this published article and its supplementary information files.

## Code Availability

https://github.com/FianaNG/autism_gene_panels.
